# Characterization of persistent headache attributed to past stroke

**DOI:** 10.1055/s-0042-1755269

**Published:** 2022-11-09

**Authors:** André Rêgo, Rita Pinheiro, Sofia Delgado, Francisco Bernardo, Elsa Parreira

**Affiliations:** 1 Hospital Professor Doutor Fernando Fonseca, Serviço de Neurologia, Amadora, Lisboa, Portugal.

**Keywords:** Vascular Headaches, Headache Disorders, Secondary, Cefaleias Vasculares, Transtornos da Cefaleia Secundários

## Abstract

**Background** Persistent headache attributed to past stroke (PHAPS) is a controversial entity, recently included in the third edition of the International Classification of Headache Disorders (ICHD-3) despite being described only in retrospective studies.

**Objective** To determine the frequency and characteristics of PHAPS in patients admitted with acute stroke.

**Methods** We selected all patients with headache associated with acute stroke (HAAS) from a prospective, single-center registry of patients with acute stroke admitted to a Neurology ward between November 2018 and December 2019. We analyzed demographic, clinical, and neuroimaging data. We assessed the follow-up with a phone call questionnaire at 6 to 12 months.

**Results** Among 121 patients with acute stroke, only 29 (24.0%) had HAAS. From these, 6 (5.0%) were lost to follow-up. In total, 23 (20.0%) patients answered the 6- to 12-month follow-up questionnaire and were included in this study. The median age of the sample was 53 years (interquartile range [IQR]: 38–78 years), and there was no sex predominance. Of the 10 patients (8,3%) that had persistent headache, 8 (6.6%) suffered from previous chronic headaches; however, they all mentioned a different kind of headache, and 1 (0,8%) probably had headache secondary to medication.

**Conclusions** In the present study, only 10 out of 121 stroke patients (8.3%) referred persistent headache at the 6- to 12-month follow-up, but the majority already suffered from previous chronic headache, which raises the question that the actual prevalence of PHAPS may be lower than previously reported.

## Resumo

**Antecedentes** A cefaleia persistente atribuída a acidente vascular cerebral (AVC) prévio é uma entidade controversa, recentemente incluída na terceira edição da Classificação Internacional de Transtornos da Cefaleia (International Classification of Headache Disorders, ICHD-3), apesar de descrita apenas em estudos restrospectivos.

**Objetivo** Determinar a frequência e as características do acidente vascular cerebral prèvio em doentes admitidos com AVC agudo.

**Métodos** Selecionamos todos os doentes com cefaleia associada a AVC agudo de um registro unicêntrico e prospectivo de doentes admitidos no serviço de Neurologia entre novembro de 2018 e dezembro de 2019. Analisamos dados demográficos, clínicos e imagiológicos. Reavaliamos aos 6 a 12 meses por questionário telefónico.

**Resultados** Entre os 121 doentes admitidos com AVC agudo, apenas 29 (24,0%) tinham cefaleia associada a AVC agudo. Destes, 6 (5,0%) perderam-se no seguimento. Ao todo, 23 (20,0%) responderam ao questionário e foram incluídos. A idade mediana foi de 53 anos (intervalo interquartílico [IIQ]: 38–78 anos), e não houve predominância de gênero. Dos 10 doentes (8,3%) que apresentam cefaleia persistente, 8 (6,6%) tinham histórico de cefaleia; no entanto todos mencionaram um tipo diferente de cefaleia, e 1 (0,8%) provavelmente tinha cefaleia secundária a medicação.

**Conclusões** Neste estudo, apenas 10 em 121 doentes (8,3%) mencionaram cefaleia persistente aos 6 a 12 meses de seguimento, mas a maioria sofria de cefaleia prévia, pelo que a real prevalência de acidente vascular cerebral prèvio pode ser inferior à descrita nos estudos realizados até a data.

## INTRODUCTION

Stroke is a very common diagnosis, with an incidence of 1.9% in the general population, and of up to 14.1% in the age group between 65 and 74 years. 1 2 Headache is a very frequent symptom, with a yearly incidence of 50% in the general population, which is why there is an important overlap of these 2 entities by mere chance. 1 2 The mechanisms behind the development of headache in stroke involve compression of pain-sensitive structures like the meninges and intracranial arteries, which does not explain the majority of the cases. 3 Other possible mechanisms concern cortical spreading depression triggered by ischemia, leading to trigeminovascular activation in cortical infarcts, with the dense trigeminovascular innervation explaining the higher prevalence of headache in posterior-circulation strokes, and also other hypotheses related to damage of pain-processing structures as well as release of inflammatory substances. 4 5 6

Persistent headache attributed to past stroke (PHAPS) is still a controversial clinical entity, with an incidence reported in the literature 6 7 8 9 between 10% and 20%. It is defined in the third edition of the International Classification of Headache Disorders (ICHD-3) as a headache fulfilling criteria for headache associated with acute stroke (HAAS) that persists after stabilization of the cerebrovascular event, and it is described for acute ischemic stroke, intracerebral hemorrhage, subarachnoid hemorrhage, acute subdural hemorrhage, carotid/vertebral dissection, and reversible cerebral vasoconstriction syndrome. 10

Previous studies 6 7 8 11 12 revealed prevalence of headache following stroke to be between 10.8% and 23.3%, and that of new headache following stroke in the range of 12%. However, most of those clinical studies were retrospective, with several limitations such as not discerning which patients had a history of primary chronic headache, not evaluating iatrogenic causes for new-onset headaches, not assessing pain medication overuse, or not even confirming the diagnosis of acute stroke through imaging studies. 6 7 8 11 12 The pathophysiology behind this presumed entity is still more obscure than the one explaining HAAS. 3

The aim of the present study was to determine the frequency and characterize PHAPS in a population of stroke patients admitted to a Neurology ward, accounting or multiple possible confounders. Our main hypothesis was that PHAPS might occur in patients predisposed to headache (those previously suffering from headache).

## METHODS

### Study design

The present is a single center prospective study. The enrolled patients or their surrogates provided written informed consent.

We estimated that 110 patients would be needed to address the primary objective, assuming an adherence of 90% to phone calls and a prevalence of 20% of headache following stroke, considering a significance level of 8% (2-sided) and a statistical power of 95%.

The study was conducted in accordance with the Declaration of Helsinki and approved by the local ethics committee.

### Patients

We selected all patients with HAAS from a prospective, single-center registry of patients with acute stroke admitted to a Neurology ward between November 2018 and December 2019.

To achieve this, every patient admitted to the Neurology ward was evaluated for inclusion in the study even without a definite diagnosis at that time. Patients with ischemic or hemorrhagic stroke confirmed by imaging methods (either computed tomography or magnetic resonance imaging scans) were included. Other inclusion criteria were being aged 18 years or older and no more than a 24-hour gap between the onset of headache and the focal symptoms (or headache alone with confirmed stroke by an imaging method in 24 hours).

Patients with communication problems (aphasia, dementia, disorders of consciousness, severe dysarthria), anosognosia, and those who did not provide consent, were pregnant or had incomplete questionnaires were excluded.

In the first days of admission, a standard questionnaire addressing the presence of headache and its characteristics was applied to all patients who had suspicion of stroke (see supplemental data). The comorbidities of the patients were extracted from the electronic registries and standard questionnaires. Important information regarding possible confounding factors for headache were included in the questionnaires.

The etiology of the stroke, for ischemic events, was determined in agreement with the Trial of Org 10172 in Acute Stroke Treatment (TOAST) classiﬁcation.

We applied questionnaires addressing possible confounders, such as depression (Patient Health Questionnaire-9, PHQ-9), sleep apnea (Epworth sleepiness scale), and the impact of headache on daily activities (Headache Impact Test-6, HIT-6). Other possible confounders were analyzed, such as the consumption of coffee or tea, weight gain, pain medication overuse, and regular medication changes following stroke.

The severity of the headache was graded as mild if the patients scored 3 or lower on the numeric rating scale (NRS), moderate, with a score between 4 and 7, and severe, with a score of 8 or higher.

The headache was classiﬁed as probable migraine (G43.83) or probable tension-type headache (G.44.28), using the ICHD-3 criteria. Patients presenting both with “probable migraine” and “probable tension-type” headache were classiﬁed as having “mixed” headache. Patients who could not be classiﬁed as having probable migraine, tension-type or mixed headache were categorized as having “other” headache.

### Follow-up

All patients with HAAS were contacted by telephone or e-mail (when available) at 6 months to a year after discharge. Several phone calls were made, and patients with 5 missed calls at different times and different days were excluded. The follow-up questionnaire can be found in the supplemental data.

### Statistical analysis

The patients were divided into two groups for statistical purposes: those who remained with headache at the follow-up questionnaire, and those who no longer complained of headache by that time. The groups were compared for medical comorbidities (including history of chronic headache), body mass index, active smoking status or history of alcohol abuse, stroke etiology, stroke location, score on the National Institutes of Health Stroke Scale (NIHSS), medication likely to cause headaches (including calcium channel blockers and dipyridamole), and other common factors known to possibly cause headache, such as depression and sleep apnea, measured using the PHQ-9 and Epworth scores accordingly.

Data were analyzed using the Statistical Package for the Social Sciences (IBM SPSS Statistics for Windows, IBM Corp., Armonk, NY, United States) software, version 23.0. The Pearson chi-squared test or Fisher exact test was conducted for the comparison of the categorical variables depending on group sizes. The *t* -test and Wilcoxon rank-sum were applied for the continuous variables. Odds ratios (ORs) were used to estimate the risk factors for developing persistent headache. Two-tailed probability ( *p* ) values < 0.05 were considered signiﬁcant.

## RESULTS

**Figure 1:**
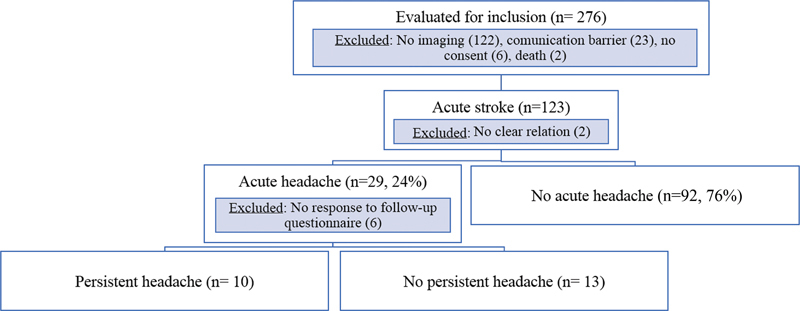
Flowchart of the study.

During the study period, 276 patients were evaluated for inclusion, and several patients were excluded according to the study protocol ( Figure 1 ). Among 121 patients with acute stroke from the registry, 29 (24.0%) had HAAS according to the ICHD-3 criteria (new acute headache that has developed in very close temporal relation to other symptoms and/or clinical signs of ischemic stroke or had led to the diagnosis of ischemic stroke). From these, 23 patients answered the 6-month follow-up questionnaire and were included in the study.

At the follow-up, both groups were similar in terms of demographic factors and clinical comorbidities. The median age was 53 years (interquartile range [IIQ]: 38.0–78.0 years), and there was no sex predominance ( Table 1 ).

**Table 1:** Population with acute stroke divided according to the presence of persistent headache

	Persistent headache	No persistent headache	p-value
Female sex: n (%)	5 (50.0%)	4 (30.8%)	0.417
Age (years): median (interquartile range)	53 (38–78)	55 (49–65)	0.852
Body mass index (kg/m ^2^ ): median (interquartile range)	29 (24–31)	25 (23–32)	0.804
Hypertension: n (%)	7 (70.0%)	9 (69.2%)	1.000
Type-2 diabetes: n (%)	3 (30.0%)	3 (23.1%)	1.000
Dyslipidemia: n (%)	5 (50.0%)	9 (69.2%)	0.417
Atrial fibrillation: n (%)	2 (20.0%)	2 (15.4%)	1.000
Chronic kidney disease: n (%)	2 (20.0%)	0 (0.0%)	0.178
Psychiatric disease: n (%)	0 (0.0%)	2 (15.4%)	0.486
Active smoking: n (%)	2 (20.0%)	4 (30.8%)	0.660
Alcohol abuse: n(%)	0 (0.0%)	2 (15.4%)	0.486
*Stroke etiology*			
Cardioembolic: n (%)	3 (30.0%)	3 (23.1%)	
Large vessel: n (%)	0 (0.0%)	0 (0.0%)	
Small vessel: n (%)	2 (20.0%)	0 (0.0%)	
Undetermined: n (%)	2 (20.0%)	4 (30.8%)	
Other ischemic etiology: n (%)	0 (0.0%)	2 (15.4%)	
Hemorrhagic stroke: n (%)	3 (30.0%)	3 (23.1%)	
Subarachnoid hemorrhage: n (%)	0 (0.0%)	1 (7.7%)	

The etiology of the stroke was similar in both groups. Patients with other etiologies (spontaneous cervical dissection and venous infarct) were only noted on the “no persistent headache” group.

In total, 10 ot of 121 (8.3%); 13 (10.7%) had persistent headache at the follow-up, and 13 (10.6%) were headache free.

Most patients suffering from persistent headache suffered from previous chronic headache. Of the 10 (8.3%), 8 (6.6%); 2 (1.7%); 1 (0.8%) patients who had persistent headache, 8 suffered from previous chronic headache; however, they all mentioned a different kind of persistent headache. Two patients had persistent headache despite not having previous chronic headache, but only 1 patient did provide other possible explanations for a secondary headache (calcium channel blockers introduced during the hospital stay). Four patients who suffered from previous chronic headache ceased to have headache complaints.

The differences in the characteristics of previous, acute and persistent headaches can be found in Table 2 .

**Table 2:** Characterization of persistent headache according to type and location

	Headache characterization			
		Previous	Acute	Persistent
Type of headache: n (%)	Migraine-type	5 (50.0%)	9 (39.1%)	1 (10.0%)
	Tension-type	4 (40.0%)	2 (8.7%)	5 (50.0%)
	Other	1 (10.0%)	12 (52.2%)	4 (40.0%)
Headache location: n (%)	Anterior	7 (70.0%)	18 (78.3%)	6 (60.0%)
	Posterior	2 (20.0%)	4 (17.4%)	2 (20.0%)
	Hemicranial	0 (0.0%)	0 (0.0%)	2 (20.0%)
	Holocranial	0 (0.0%)	1 (4.3%)	0 (0.0%)
	Bilateral	7 (70.0%)	16 (69.6%)	5 (50.0%)
	Unilateral	2 (20.0%)	6 (26.1%)	4 (40.0%)
	Ipsilateral	−	6 (26.1%)	2 (20.0%)

**Table 3:** Univariate logistic regression

Univariate logistic regression	Odds ratio	95% confidence interval	*p* -value
History of chronic headache	9.000	1.285–63.025	0.027
Arterial territory	0.429	0.073–2.500	0.346
Cortical stroke	0.857	0.164–4.467	0.855
NIHSS score upon admission	0.816	0.573–1.162	0.259
Calcium channel blockers at discharge	0.964	0.160–5.795	0.968
Epworth score at follow-up	0.930	0.718–1.206	0.586
Score≥8 on the PHQ-9 questionnaire at follow-up	8.000	0.725–88.226	0.090

Abbreviations: NIHSS, National Institutes of Health Stroke Scale; PHQ-9, Patient Health Questionnaire-9.

The type of headache was more frequently migraine or tension-type before stroke, but acute headache was mainly of the migraine type or had mixed characteristics. Persistent headache was more of the tension type or had mixed characteristics.

The location of the pain was more frequently anterior and bilateral in all types of headache, but unilateral persistent headache was not typically ipsilateral to stroke, as opposed to acute headache. Pain was mild to moderate in most cases ( *n* = 7/10, 70.0%). Most patients ( *n* = 6/10, 60.0%) had fewer than 2 episodes a month. Only 2 ( *n* = 2/10, 20.0%) patients referred highly frequent episodes of 15 days a month.

On the univariate logistic regression ( Table 3 ), we observed a statistically significant association between the presence of previous chronic headache and persistent headache (univariate analysis with an OR of 17.623 [range: 1.287–244.374]; *p* = 0.027). The analysis also showed a trend toward a possible association between persistent headache and depressive symptoms (univariate analysis with an OR of 8.000 [range: 0.725–88.226]; *p* = 0.090).

## DISCUSSION

The main finding in our prospective cohort was that previous chronic headache was the only factor associated with persisting headache after stroke, although there was a change in the usual headache pattern. Interestingly, 4 (3.3%) patients who suffered from previous chronic headache even ceased to have headache complaints. This finding is corroborated by the fact that persistent headache complaints were only ipsilateral to stroke in a minority of patients, as opposed to what is observed in the acute phase of stroke.

In the present study, only 1 patient seemed to have a persistent headache *de novo* , without a history of chronic headache or other causes for new-onset headache, corresponding to a very low percentage (< 0.8%) when compared with other previous studies and similar to novel headache in reference subjects without stroke, serving as a control group, and also similar to non-stroke populations. 13 14 As such, with the present study, we may question the existence of this new entity.

Little is known about the molecular and metabolic changes that occur after stroke, but there is evidence of some network reorganization with recruitment of neighboring and contralateral areas with similar functions as well as increased frontal lobe connectivity, possibly reflecting executive back-up strategies for te replacement of lost functions. 15 16 Recent studies 17 18 19 20 21 22 23 24 showed a complex network on migraine pathophysiology with altered connectivity scattered through the brain (cortex, thalamus, hypothalamus, brainstem, amygdala, and cerebellum). Likewise, in tension-type headache, structural abnormalities have been shown along the pain matrix. 25 Disruption of the pain modulation network may explain the changes in headache patterns and, ultimately, explain total headache remission.

We excluded many patients (n = 122) for several reasons. First of all, we aimed to apply questionnaires to every patient with acute stroke, even if the likelihood of stroke diagnosis was low, resulting in a high number of questionnaires perfomed to stroke mimics; we also excluded patients with clinical strokes but without magnetic resonance imaging on follow-up either due to low clinical value of performing this complementary work-up but also due to this being performed long after the acute phase of stroke.

Lesion size was not analyzed because of the wide variability in stroke etiology, but the NIHSS score upon admission may have served as a potential surrogate for this factor.

The sample size did not enable us to draw conclusions regarding a possible association between the etiology of the stroke and the persistence of the headache, as opposed to what has been extensively noted in acute-phase headache. 8 26 27 28 29 30 31 32 33 34 35 36 37 38 39 40

The strength of the present study was clearly defining which patients already suffered from chronic headache upon admission, as this is a common complaint in the general population which has never been assessed in previous similar studies. One of the biggest weaknesses was the heterogeneity in stroke etiology, with the inclusion of mainly ischemic strokes, but also hemorrhagic strokes and subarachnoid hemorrhages, which may influence tissue reorganization in different ways.

In conclusion, in the present single-center prospective study, although 10 out of 121 stroke patients (8.3%) mentioned persistent headache at follow-up, most already suffered from chronic headache, which raises the hypothesis that the actual prevalence of PHAPS may be lower than previously reported. With the present study, we may even question the existence of this recently-described clinical concept.
